# Shared genetic risk between migraine and coronary artery disease: A genome-wide analysis of common variants

**DOI:** 10.1371/journal.pone.0185663

**Published:** 2017-09-28

**Authors:** Bendik S. Winsvold, Francesco Bettella, Aree Witoelar, Verneri Anttila, Padhraig Gormley, Tobias Kurth, Gisela M. Terwindt, Tobias M. Freilinger, Oleksander Frei, Alexey Shadrin, Yunpeng Wang, Anders M. Dale, Arn M. J. M. van den Maagdenberg, Daniel I. Chasman, Dale R. Nyholt, Aarno Palotie, Ole A. Andreassen, John-Anker Zwart, The International Headache Genetics Consortium

**Affiliations:** 1 FORMI and Department of Neurology, Oslo University Hospital, Oslo, Norway; 2 Institute of Clinical Medicine, University of Oslo, Oslo, Norway; 3 NORMENT KG Jebsen Centre, Division of Mental Health and Addiction, Oslo University Hospital, Oslo, Norway; 4 Analytic and Translational Genetics Unit, Massachusetts General Hospital and Harvard Medical School, Boston, Massachusetts, United States of America; 5 Stanley Center for Psychiatric Research, Broad Institute of MIT and Harvard, Cambridge, Massachusetts, United States of America; 6 Program in Medical and Population Genetics, Broad Institute of MIT and Harvard, Cambridge, Massachusetts, United States of America; 7 Psychiatric & Neurodevelopmental Genetics Unit, Department of Psychiatry Massachusetts General Hospital, Boston, Massachusetts, United States of America; 8 Institute of Public Health, Charité–Universitätsmedizin Berlin, Berlin, Germany; 9 Division of Preventive Medicine, Brigham and Women’s Hospital, Harvard Medical School, Boston, Massachusetts, United States of America; 10 Department of Neurology, Leiden University Medical Centre, Leiden, The Netherlands; 11 Department of Neurology and Epileptology, Hertie-Institute for Clinical Brain Research, University of Tübingen, Tübingen, Germany; 12 Institute for Stroke and Dementia Research, Klinikum der Universität München, Ludwig-Maximilians-Universität München, Munich, Germany; 13 Center for Multimodal Imaging & Genetics, University of California, San Diego, La Jolla, California, United States of America; 14 Department of Human Genetics, Leiden University Medical Centre, Leiden, The Netherlands; 15 Harvard Medical School, Boston, Massachusetts, United States of America; 16 Statistical and Genomic Epidemiology Laboratory, Institute of Health and Biomedical Innovation, Queensland University of Technology, Kelvin Grove, Australia; Kunming Institute of Zoology, Chinese Academy of Sciences, CHINA

## Abstract

Migraine is a recurrent pain condition traditionally viewed as a neurovascular disorder, but little is known of its vascular basis. In epidemiological studies migraine is associated with an increased risk of cardiovascular disease, including coronary artery disease (CAD), suggesting shared pathogenic mechanisms. This study aimed to determine the genetic overlap between migraine and CAD, and to identify shared genetic risk loci, utilizing a conditional false discovery rate approach and data from two large-scale genome-wide association studies (GWAS) of CAD (C4D, 15,420 cases, 15,062 controls; CARDIoGRAM, 22,233 cases, 64,762 controls) and one of migraine (22,120 cases, 91,284 controls). We found significant enrichment of genetic variants associated with CAD as a function of their association with migraine, which was replicated across two independent CAD GWAS studies. One shared risk locus in the *PHACTR1* gene (conjunctional false discovery rate for index SNP rs9349379 < 3.90 x 10^−5^), which was also identified in previous studies, explained much of the enrichment. Two further loci (in *KCNK5* and *AS3MT*) showed evidence for shared risk (conjunctional false discovery rate < 0.05). The index SNPs at two of the three loci had opposite effect directions in migraine and CAD. Our results confirm previous reports that migraine and CAD share genetic risk loci in excess of what would be expected by chance, and highlight one shared risk locus in *PHACTR1*. Understanding the biological mechanisms underpinning this shared risk is likely to improve our understanding of both disorders.

## Introduction

Migraine is a recurrent pain condition which affects some 14% of the general population and is ranked as the 7^th^ leading cause of disability worldwide [1–3]. In about one third of patients, headache attacks are preceded by transient neurological symptoms, termed migraine aura [2]. The pathogenic mechanisms of migraine are incompletely understood, but the disorder has a considerable genetic component with an estimated heritability of 42% [4]. There has been a long-standing debate over whether migraine is primarily a disorder of vascular or neuronal origin, but the more recent view favors a neuronal origin [5, 6]. Several arguments exist, however, why a partly vascular pathology in migraine should not be dismissed. First, migraine is associated with a range of vascular disorders [7], most notably, individuals with migraine have a two-fold increased risk of experiencing an ischemic stroke, a risk that is most apparent for migraine with aura [8]. More recently, a similar risk increase has been reported for coronary artery disease (CAD) suggesting a relation between migraine and vascular pathology outside the cerebral circulation [9, 10]. Second, migraine patients more often report a family history of early CAD, suggesting a possible shared genetic basis between both disorders [11]. Last, genetic loci associated with migraine appear to be enriched for genes expressed in vascular and smooth muscle tissues, pointing towards vascular mechanisms [12].

Recent large-scale genome-wide association studies (GWAS) allow for the interrogation of shared genetic risk factors between traits [13]. If migraine and CAD have a partly shared pathogenic basis, it is likely that they also share genetic risk factors, which has indeed been found for migraine and stroke [14] and migraine and CAD [15, 16], respectively. Here we aimed to further investigate the polygenic overlap between migraine and CAD, and to identify common susceptibility gene loci pointing to shared mechanisms in both disorders. To this end we made use of three large-scale GWAS of CAD and migraine, together with a newly developed method that utilizes a conditional false discovery rate (FDR) approach. This approach has been used to identify shared risk loci and potential novel susceptibility variants for a number of diseases and phenotypes, including schizophrenia [17, 18], bipolar disorder [17], prostate cancer [19], hypertension [20], primary sclerosing cholangitis [21], and Alzheimer’s disease [22].

## Materials and methods

### Participant samples

**Fig 1:**
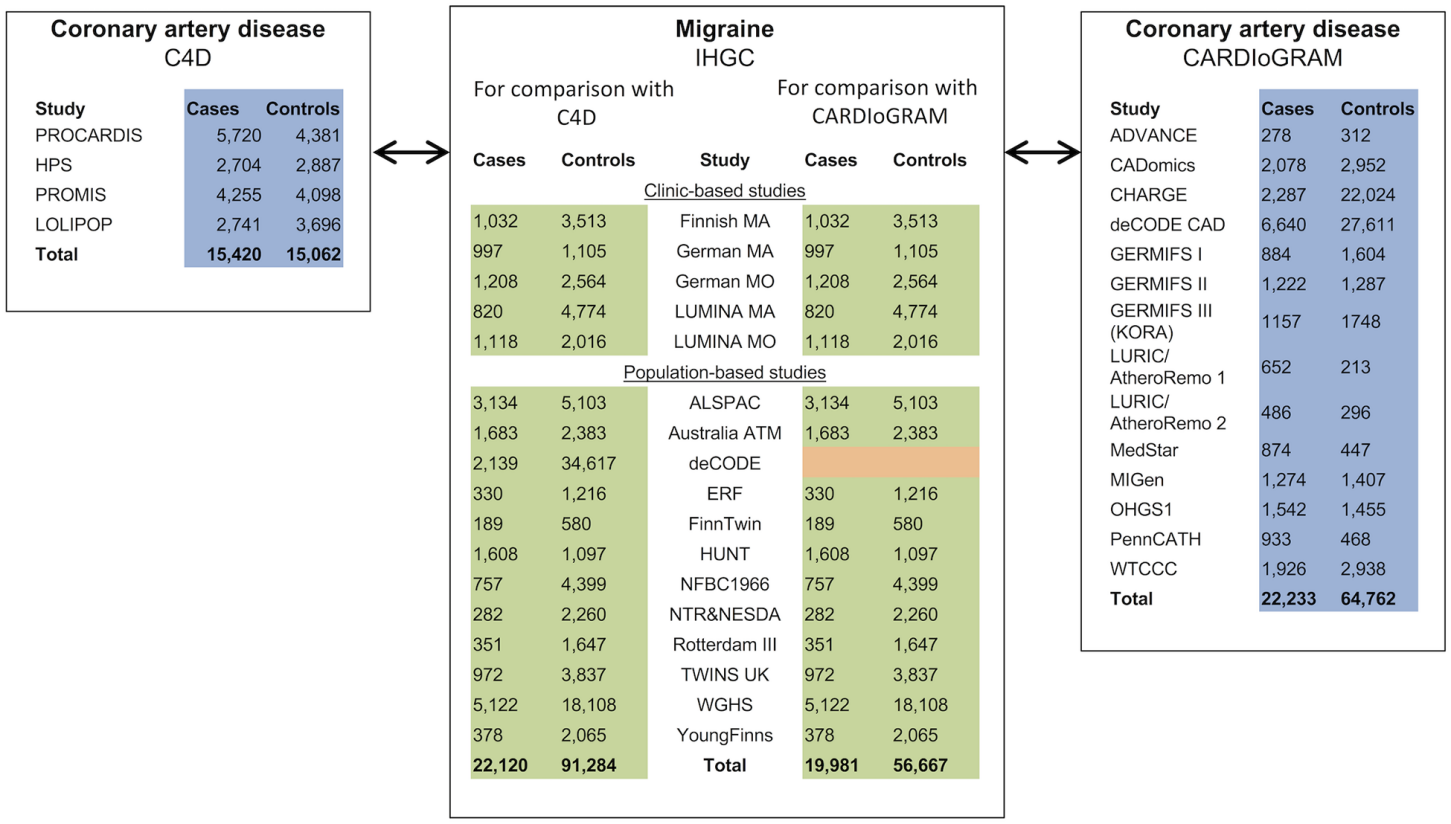
Overview of the included studies.

Analyses are based on three large GWAS meta-analyses of migraine and CAD (Fig 1).

Details about recruitment, phenotyping, genotyping and association analyses are provided in the original publications [23–25]. Migraine GWAS summary statistics results (P-values and z-scores) were obtained from the International Headache Genetics Consortium (IHGC) [24] and entailed 23,285 migraine cases and 95,425 controls. Through public-access websites, we obtained the summary statistics for two GWAS of CAD, C4D [23] with 15,420 cases and 15,062 controls, and CARDIoGRAM [25] with 22,233 cases and 64,762 controls. To achieve independence between the migraine and CAD datasets we re-performed the IHGC migraine meta-analysis without the 1958 Birth Cohort (B58C) (thereby excluding 1,165 cases and 4,141 controls), leaving the migraine and C4D datasets without overlapping samples. For comparison with CARDIoGRAM we re-performed the migraine analysis without B58C and deCODE cohorts (thereby excluding 3,304 cases and 38,758 controls). This left an overlap of 834 controls from GERMIFS III (KORA), which was also part of the German MO migraine sub-study. This overlap was deemed minimal (representing < 1.5% of the controls in the CARDIoGRAM and migraine studies). As excluding the German MO cohort (the largest clinical migraine cohort) would substantially reduce the overall power of the migraine analysis, this cohort was kept in the analysis. This resulted in two migraine datasets with 22,120 and 19,981 cases, and 91,284 and 56,667 controls, for comparison with C4D and CARDIoGRAM, respectively. The two CAD GWAS studies, C4D and CARDIoGRAM, contained no overlapping samples.

The study was approved by the Regional Committee for Ethics in Medical Research, Norway (#2012/229/REK sor-ost C). The relevant institutional review boards or ethics committees approved the research protocol of the individual GWAS studies used in the current analysis, and all human participants gave written informed consent.

### Statistical analyses

#### Conditional Q-Q plots for cross-phenotype enrichment

**Fig 2:**
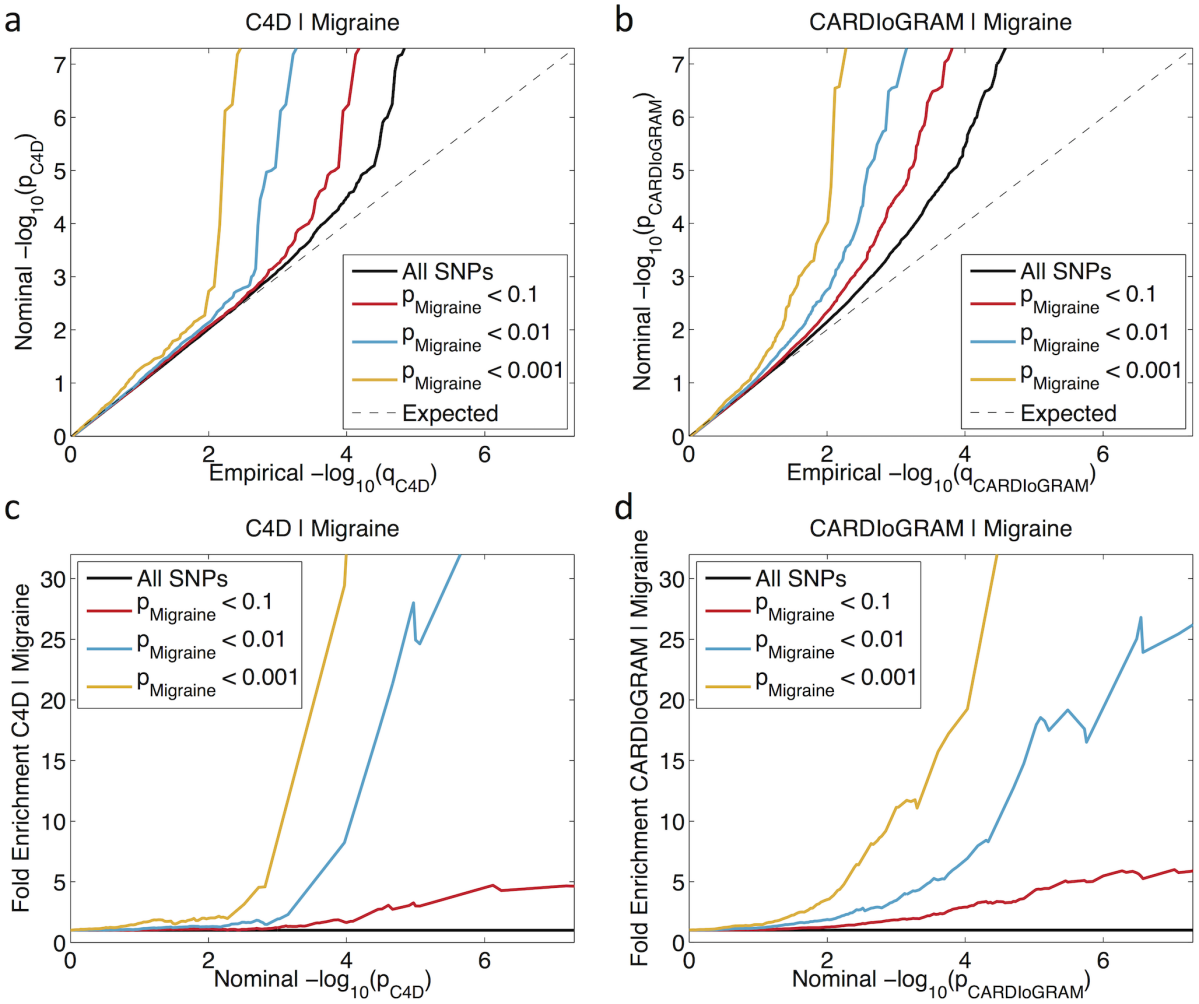
Genetic cross-phenotype enrichment of CAD conditional on migraine. (**a-b**) Conditional Q-Q plot of nominal versus empirical -log_10_ P-values (corrected for inflation) in CAD as a function of significance of association with migraine at the level of P ≤ 1, P < 0.1, P < 0.01 and P < 0.001. Dotted lines indicate the null-hypothesis. (**c-d**) Plots showing fold enrichment for association to CAD in a given -log_10_ P-value bin as a function of association with migraine.

To visually assess cross-phenotype enrichment, we used modified quantile-quantile (Q-Q) plots for association to CAD conditioned on ‘cross-phenotype' effects (Fig 2a and 2b), described in detail previously [17, 18].

We say cross-phenotype ‘enrichment’ exists between CAD and migraine if the degree of deflection of the CAD Q-Q line from the expected null line is dependent on the reliability of the associations with migraine. We first applied a genomic control method using only intergenic single nucleotide polymorphisms (SNPs) to compute the inflation factor, λ_GC_, and divided all test statistics by λ_GC_ as detailed in previous publications [17, 18, 20]. Next, we constructed conditional Q-Q plots of empirical versus nominal cumulative −log_10_(P) values quantiles for all SNPs and for different subsets of SNPs determined by the significance of their association with migraine. Specifically, we computed the empirical cumulative distribution of nominal −log_10_(P) values for CAD for all SNPs and for SNPs with significance levels below the indicated cut-offs for migraine (P < 0.1, P < 0.01, P < 0.001). The nominal P-values (−log_10_(p)) are plotted on the y-axis, and the empirical quantiles (−log_10_(q), where q = 1-cdf(p)) are plotted on the x-axis. To assess polygenic effects below the standard GWAS significance threshold, we focused the Q-Q plots on SNPs with nominal −log_10_(p) < 7.3 (corresponding to P > 5x10^-8^). The information contained in the Q-Q plots can equivalently be reported in the form of fold enrichment plots (Fig 2c and 2d) which emphasizes how much more likely the selected SNPs fall in each cumulative bin compared to the baseline. The ‘enrichment’ seen in the conditional Q-Q plots can be directly interpreted in terms of true discovery rate (TDR = 1 − FDR) [26]. The analysis was repeated independently for comparison of C4D against migraine and for CARDIoGRAM against migraine.

#### Conjunction statistics—Test of association with both phenotypes

To detect loci showing strong evidence of association with both CAD and migraine, we used a conjunction statistical testing procedure as described in detail previously [18]. We first calculated conditional FDRs, defined as the posterior probability that a given SNP is null for the first phenotype given that the P-values for both phenotypes are as small as or smaller than their observed P-values. We assigned a conditional FDR value for each SNP in migraine given its P-value in CAD (denoted as FDR _MIG|CAD_), and in CAD given its P-value in migraine (FDR_CAD|MIG_). Using these, we constructed a bidirectional 2D conjunctional FDR look-up table (FDR_MIG & CVD_) computing the maximum conditional FDR in both directions, i.e.,

$${\text{FDR}}_{\text{MIG}\;\&\;\text{CVD}}=\text{max}\left({\text{FDR}}_{\text{MIG}|\text{CVD}}{,\;\text{FDR}}_{\text{CVD}|\text{MIG}}\right).$$

Subsequent interpolation of the look-up table yields the desired conjunction statistic. By down-weighting single phenotype effects driving the common association signal, the conjunction statistic allows the identification of SNPs that are more likely to be associated with both phenotypes. To illustrate the localization of the identified cross-phenotype SNPs, we used a 'Conjunction Manhattan plot', for which we plotted the −log_10_(conjunctional FDR) against the chromosomal location for all SNPs. The strongest representative from each linkage disequilibrium (LD) block was identified by ranking all SNPs in increasing order, based on the conjunctional FDR and then removing SNPs in LD *r*^*2*^ > 0.1 with any higher-ranked SNP, according to the 1000 genomes LD structure [27]. The resulting SNPs are thus the ones deemed most significant in their respective LD blocks, all of which can be taken to represent separate loci (numbered loci). Genes close to each SNP were obtained from the NCBI gene database. A conjunctional FDR < 0.05 was considered statistically significant.

#### Random LD-pruning

In order to partially account for the physiological SNP correlation bias due to LD, all statistics illustrated above were repeatedly carried out on 100 sets of near-independent SNPs. These were obtained by randomly selecting representatives from all LD-blocks consisting of SNPs with pairwise LD *r*^*2*^ > 0.1 within 1 megabase (Mb) from one another.

#### DEPICT genetic enrichment analysis

To examine whether loci with evidence for cross-phenotype association showed enrichment for specific biological pathways or tissues, we used the DEPICT computational tool which utilizes data from 37,427 human microarray samples for 209 tissue or cell types [28]. The analysis took as input independent SNPs with conjunctional FDR > 0.4 (PLINK clumping parameters: -clump-p1 0.4—clump-r2 0.5 clump-kb 250), corresponding to 114 SNPs comprising 192 genes for the comparison of migraine against CARDIoGRAM and 41 SNPs comprising 75 genes for migraine against C4D. DEPICT was run using default settings.

#### Characterization of identified cross-phenotype association loci

Effects of the cross-phenotype association loci on regional gene expression (cis effect) were examined using the Genotype-Tissue Expression (GTEx) database, a high quality database of gene expression across various human tissues [29]. Expression quantitative trait loci (eQTLs) within a +/- 1Mb *cis* window around the transcription start site for each transcript were pre-calculated in the GTEx project, with significance determined using a Q-value threshold [29]. Previously reported genome-wide significant associations (P-value < 5 x 10^−8^) at each locus were identified by considering SNPs in LD with the index SNP (*r*^*2*^ > 0.1 in European CEU, Phase 3 of the 1000 Genomes Project [30]) represented in the NHGRI GWAS catalog [31].

## Results

### Enrichment of shared associations between migraine and CAD

Stratified Q-Q plots for CAD conditioned on nominal P-values of association to migraine show cross-phenotype enrichment across different levels of significance for CAD (Fig 2a and 2b). Similar results were observed for C4D and CARDIoGRAM. The earlier departure from the null line (leftward shift) indicates a greater proportion of true associations for a given nominal CAD P-value. Successive leftward shifts for decreasing nominal migraine P-values indicate that the proportion of non-null effects varies considerably across different levels of association with migraine. For example, in the migraine P-value < 0.001 category, the proportion of SNPs reaching -log_10_(P_CAD_) > 4 is more than 30 times greater than the −log_10_(P_CAD_) ≥ 0 category (all SNPs) for C4D (Fig 2c), and about 20 times greater for CARDIoGRAM (Fig 2d), indicating a high level of enrichment.

### Cross-phenotype gene loci in migraine and CAD

**Fig 3:**
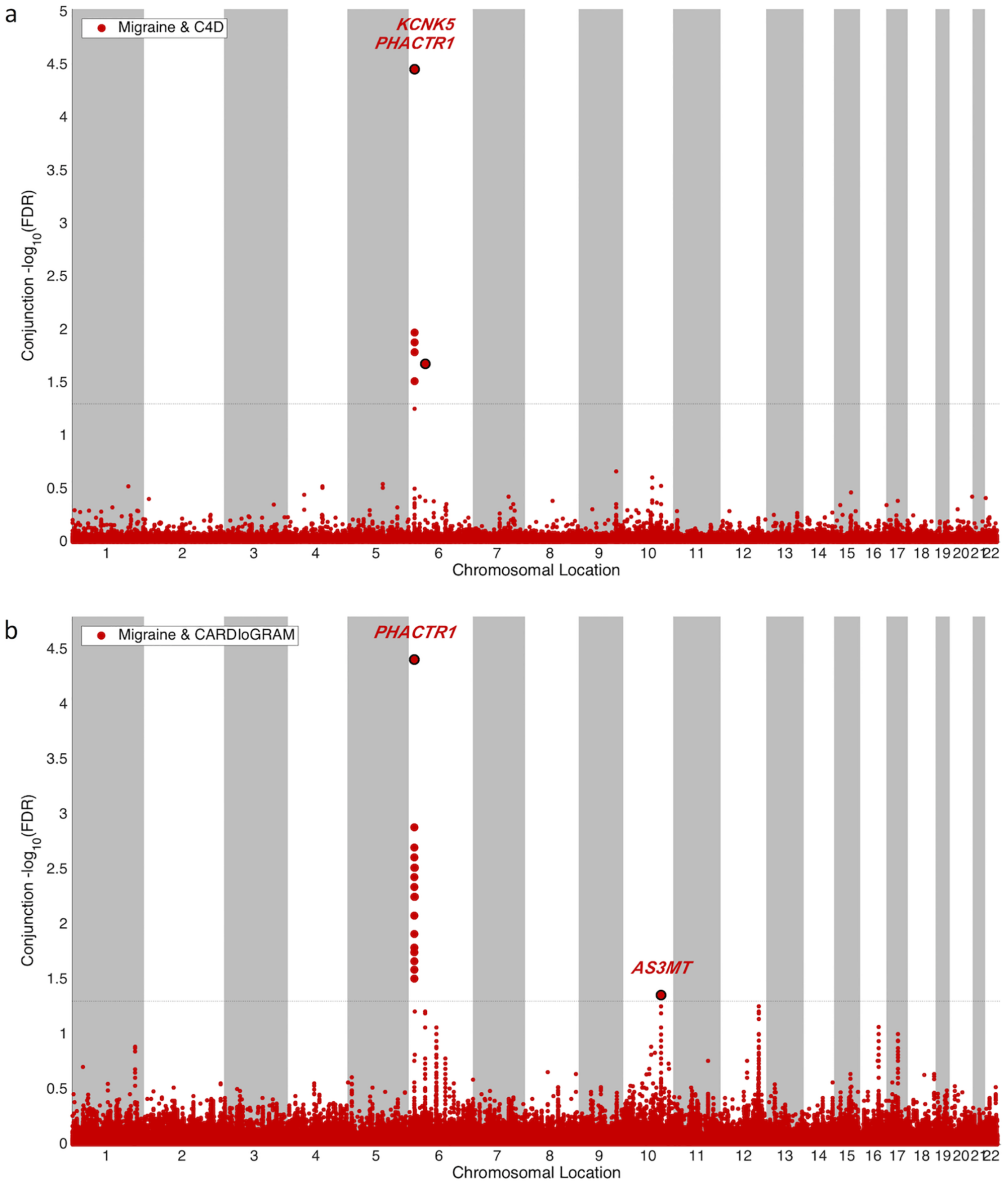
Conjunction FDR Manhattan plots—Shared risk loci between migraine and coronary artery disease (CAD). SNPs with conjunctional false discovery rate (FDR) < 0.05 are shown with large points. A black line around the large points indicate the most significant SNP in each linkage disequilibrium block, annotated with the closest gene. Separate plots are shown for cross-phenotype loci between **a**) migraine and C4D, and **b**) migraine and CARDIoGRAM.

An overlap of genetic risk factors between migraine and CAD may represent shared pathological mechanisms between both disorders. To provide an unselected map of shared risk loci between migraine and CAD, we performed a conjunction-FDR analysis. Three independent loci, located on chromosomes 6 and 10, showed significant cross-phenotype association (conjunctional FDR for index SNP < 0.05; Table 1, Fig 3).

**Table 1:** SNPs showing significant evidence (conjunctional FDR < 0.05) for shared association to migraine and coronary artery disease (CAD).

Locus	Index SNP	Chr	Position*	Nearest Gene	Effect allele	Migraine beta (SE)	Migraine P-value	CAD beta (SE)	CAD P-value	Conjunctional FDR
Comparison Migraine and CAD: C4D										
Locus 1	rs9349379	6	13011943	*PHACTR1*	A	0.073 (0.014)	6.44E-08	-0.159 (0.017)	6.50E-21	3.50E-05†
Locus 2	rs733701	6	39279840	*KCNK5*	T	0.058 (0.014)	2.24E-05	0.075 (0.019)	5.86E-5	0.021†
Locus 3	rs10786719	10	104627982	*AS3MT*	A	na	na	na	na	na
Comparison Migraine and CAD: CARDIoGRAM										
Locus 1	rs9349379	6	13011943	*PHACTR1*	A	0.083 (0.014)	7.23E-09	-0.093 (0.018)	1.54E-7	3.90E-05†
Locus 2	rs733701	6	39279840	*KCNK5*	T	0.057 (0.014)	7.10E-05	0.057 (0.016)	4.00E-04	0.065
Locus 3	rs10786719	10	104627982	*AS3MT*	A	-0.048 (0.013)	1.49E-04	0.052 (0.014)	2.08E-4	0.044†

SNP, Single nucleotide polymorphism; Chr, Chromosome; CAD, Coronary artery disease; FDR, False discovery rate. SE, standard error. na, SNP not available for analysis.

*Positions refer to build NCBI36/hg18.

^†^Conjunctional FDR < 0.05.

Details of the index SNP at each locus with conjunctional FDR < 0.1 are given in S1 Table. Locus 1 is intragenic in *PHACTR1* (encoding phosphatase and actin regulator 1 protein), and is the strongest shared risk locus between migraine and CAD in the analyses based on either C4D or CARDIoGRAM (conjunctional FDR for index SNP = 3.50 x 10^−5^ and 3.90 x 10^−5^, respectively). The top SNP rs9349379 is associated with the expression of *PHACTR1* in various arterial tissues (Table 2). Locus 2 is intragenic in *KCNK5*, a gene encoding a two-pore domain potassium channel (conjunctional FDR = 0.021 and 0.065 in C4D or CARDIoGRAM, respectively). Locus 3 is intragenic in *AS3MT*, that encodes arsenite methyltransferase. The locus shows cross-phenotype association between migraine and CARDIoGRAM (conjunctional FDR for index SNP = 0.044; not available in C4D). Top SNP rs10786719 is an eQTL for AS3MT in heart and adrenal gland, and in cerebellum for nearby gene *ARL3* (ADP-Ribosylation Factor-Like Protein 3), the SNP being located 204 kb upstream from this gene (Table 2). Previously reported genome-wide significant associations at each of the three loci are given in S2 Table.

**Table 2:** Expression quantitative trait loci (eQTLs) at the identified cross-phenotype loci.

Locus	Index SNP	eQTL gene	eQTL tissue	eQTL P-value
Locus 1	rs9349379	*PHACTR1*	Artery—Tibial	7.2E-16
		*PHACTR1*	Artery—Aorta	4.3E-12
		*PHACTR1*	Artery—Coronary	8.6E-7
Locus 3	rs10786719	*AS3MT*	Adrenal Gland	4.4E-16
		*AS3MT*	Heart—Left Ventricle	1.2E-11
		*AS3MT*	Heart—Atrial Appendage	3.4E-8
		*ARL3*	Brain—Cerebellum	1.4E-5

Single-tissue eQTLs from GTEx database. Only eQTLs with RefSeq genes are shown.

SNP, Single nucleotide polymorphism; eQTL, Expressive quantitative-trait locus.

The DEPICT genetic enrichment analysis did not identify specific tissues or biological gene set categories significantly enriched for genes in cross-phenotype loci (S1 and S2 Figs).

As a *post hoc* analysis, to investigate to what extent locus 1 (in *PHACTR1*) was driving the enrichment of cross-phenotype associations between migraine and CAD, we re-performed the enrichment analysis after excluding all SNPs in *PHACTR1* as well as any SNPs in LD (*r*^*2*^ > 0.1) with these. The resulting enrichment plots were markedly attenuated showing some residual enrichment between migraine and CARDIoGRAM, but not between migraine and C4D (S3 Fig).

## Discussion

In this large study based on data from 59,773 subjects with migraine or CAD we show that genetic variants associated with migraine are also associated with CAD in excess of what would be expected by chance. This enrichment replicated across two independent CAD GWAS studies. Three loci, in *PHACTR1*, *KCNK5* and *AS3MT*, showed significant cross-phenotype association.

Our results are in line with previous findings that migraine and CAD share genetic risk factors [15, 16]. One of these studies used the same migraine dataset as in the current study, but only one of the CAD datasets (CARDIoGRAM) [15]. The other study was based on an independent migraine dataset (23andMe) [16]. The current study also differs from previous reports by using a novel analytical approach developed for polygenic phenotypes—the stratified conditional-FDR method [32].

The three identified loci were all intragenic. Two have previously been identified as cross-phenotype loci between migraine and CAD (locus 1 in *PHACTR1* and locus 3 in *AS3MT*, Table 1) [15, 16, 24], while one (locus 2, in *KCNK5*) is new. *Post hoc* analysis showed that much of the cross-phenotype enrichment was driven by the *PHACTR1* locus. The index SNPs at two of the three loci (locus 1 in *PHACTR1* and locus 3 in *AS3MT*) show opposite effect directions in migraine and CAD, which corresponds with recent reports that CAD risk alleles overall are under-represented in migraine patients [15], and that migraine risk alleles are under-represented in CAD [16]. A GWAS of cervical artery dissection identified genome-wide association at the exact same index SNP rs9349379 in *PHACTR1* as in the current study, with its effect in the same direction as for migraine but opposite of CAD [33]. In that study, two additional established migraine risk variants, rs11172113 in *LRP1* and rs13208321 in *FHL5*, showed suggestive evidence for association to cervical artery dissection, with the same effect direction as for migraine. The latter was subsequently reported as a cross-phenotype locus between migraine and CAD with opposite effect directions [15]. From epidemiological studies there is a known co-morbidity between migraine and both cervical artery dissection [34] and CAD [9, 10], and it is intriguing that some of the same genetic variants seem to be involved in all three disorders, but with partly opposite effect directions. Variants with opposite effect direction cannot directly explain the comorbidity between migraine and CAD. However, such variants may also be of interest as they can give information on how tilting the relevant biological system in a specific direction can predispose to one disorder while protecting against the other (see e.g. [35]) or point to regulatory hubs [36]. Also, since the associated risk variants are not necessarily the causal ones, interpretation of directionality may be confounded by haplotype effects, as well as heterozygous advantage, tissue-specificity, or interactions with other genetic variants or environmental factors [37]. A better understanding of the mechanisms underpinning these shared genetic associations could shed light on vascular mechanisms in migraine and give further insight into the pathogenic basis of both disorders.

Several methods have been developed to study cross-phenotype genetic associations [13, 36]. One newly developed approach is linkage disequilibrium (LD) score regression [38]. This method estimates shared heritability between traits based on GWAS summary statistic results, and appears robust to overlapping samples between studies, thus conveniently allowing cross-phenotype analysis using readily available GWAS results. A limitation of this and other methods that focus on shared heritability is that their power is proportional to the overall correlation between the effects on the two traits under investigation [36]. For example, the LD-score regression has not found any significant amount of heritability to be shared between migraine and CAD [16]. The statistical approach applied in the current study is insensitive to the extent of the overall correlation between the effects on the respective traits and relies only on the association with these. Such balanced correlation-anticorrelation scenarios may also be of biological interest, as discussed above.

We hypothesize that genes indicated by shared loci may point to biological mechanisms shared between migraine and CAD. The strongest shared risk locus was intragenic in *PHACTR1*. This is a recognized genome-wide significant risk locus for both migraine and CAD [23, 25, 39]. The gene product of *PHACTR1*, Phosphatase And Actin Regulator 1 protein, is highly expressed in the brain where it regulates synaptic activity and dendritic morphology [40]. It is also expressed in arteries and has a role in the regulation of endothelial function and is associated with altered vasomotor tone [29, 41, 42]. As shown in Table 2, the index SNP rs9349379 is associated with the expression of *PHACTR1* in several arterial tissues. Both endothelial and vasomotor dysfunctions have been implicated in migraine [7, 43], and the locus is an attractive focus for future experimental studies to investigate shared pathogenic mechanisms between migraine and vascular disease. Locus 2 (intragenic in *KCNK5*) was recently established as a genome-wide significant locus for migraine [12]. The transcript of *KCNK5* (Potassium Channel, Two-Pore Domain Subfamily K, Member 5) is a two-pore domain potassium channel which is highly expressed in kidney [44]. Its expression in the central nervous system is limited to certain brainstem nuclei and the cochlea, where its role includes chemosensitivity to oxygen in the central control of respiration [44, 45]. Its potential role in migraine or vascular disease is unclear, but as it is involved in setting the membrane potential of pulmonary artery myocytes [46], a possible role in vasomotor regulation can be hypothesized. Locus 3 is intragenic in *AS3MT*, which encodes Arsenite Methyltransferase. A different SNP (rs7085104) at this locus is associated with schizophrenia, and has been shown to act through the up-regulation of a human-specific truncated isoform of *AS3MT* which is expressed in neurons and astrocytes [47]. Our index SNP rs10786719 is in moderate LD with rs7085104 in Europeans (r^2^ = 0.64) [30], and it possible that it could act through the same truncated isoform of AS3MT. rs10786719 is also in LD with reported risk SNPs for several vascular traits: Cerebral white matter hyperintensity burden [48], coronary heart disease and intracranial aneurysm [25, 49], blood pressure [50], and body mass index [51] (S2 Table). The potential pathogenic mechanism of the locus in migraine or vascular disease remains an enigma. Three further loci showed suggestive evidence for shared association (conjunctional FDR < 0.1, S1 Table), one of these (locus 5 near *BCAR1*) was previously identified as a cross-phenotype locus between migraine and CAD [16]. Gene set enrichment analysis of a larger set of suggestive cross-phenotype loci did not reveal enrichment in specific tissues or pathways. This is likely a consequence of the low number of cross-phenotype loci identified.

Strengths of this study are: (i) the use of three large-scale GWAS of migraine and CAD; (ii) the use of a statistical approach that allowed the identification of cross-phenotype enrichment independent of effect direction concordance, and that can pinpoint shared risk loci; and (iii) that the results were replicated across two independent CAD studies. Certain limitations should also be acknowledged: (i) while we demonstrate enrichment for cross-phenotype associations between migraine and CAD, further experimental evidence will be needed to understand the biological mechanisms underpinning the identified cross-phenotype loci; (ii) GWAS studies of migraine have been successful in identifying risk loci, but most of the associations seem to be driven by migraine without aura, and no robustly replicable loci have so far been established for migraine with aura [12, 24]. For this reason we focused the analysis on migraine overall, rather than on its subtypes. However, it can be envisaged that future studies will unravel specific genetic risk factors for migraine subtypes, which would allow sub-type-specific cross-phenotype analysis against CAD.

In conclusion, we show that genetic variants associated with migraine are also associated with CAD, and that this enrichment replicates across two independent CAD studies. Much of the enrichment was explained by one shared risk locus in *PHACTR1*, in which the index SNP affects the risk for migraine and CAD in opposite direction. The results reaffirm previous reports, in addition to suggesting a novel shared risk locus in *KCNK5*. A better understanding of the biological mechanisms underpinning shared genetic risk loci may improve our understanding of pathogenic mechanisms and shed light on vascular mechanisms in migraine.

## Supporting information

S1 Fig DEPICT analysis of gene expression enrichment in specific tissues. The analysis was based on expression data obtained from 37,427 human microarray samples for 209 tissue or cell types, as implemented in DEPICT [28]. Genes in loci with conjuctional FDR < 0.4 for cross-phenotype association between migraine and CAD were assessed for high expression in each of the annotation categories. The figures show the most enriched tissue types for the comparison of migraine against CARDIoGRAM (**a**) and for migraine against C4D (**b**). No tissue type was significantly enriched (false discovery rate <0.05) after controlling for multiple testing.
(PDF)

S2 Fig DEPICT analysis of gene set enrichment. DEPICT reconstituted gene sets showing strongest evidence for enrichment for genes in loci with conjuctional FDR < 0.4 for cross-phenotype association between migraine against CARDIoGRAM (**a**) and for migraine against C4D (**b**). No reconstituted gene sets was significantly enriched (false discovery rate <0.05) after controlling for multiple testing.
(PDF)

S3 Fig Genetic cross-phenotype enrichment of migraine conditional on CAD after excluding PHACTR1. Genome-wide analysis after excluding all SNPs in *PHACTR1* as well as any SNPs in linkage disequilibrium (*r*^*2*^ > 0.1) with these. (**a-b**) Conditional Q-Q plot of nominal versus empirical -log_10_ P-values (corrected for inflation) in CAD as a function of significance of association with migraine at the level of P ≤ 1, P < 0.1, P < 0.01 and P < 0.001. Dotted lines indicate the null-hypothesis. (**c-d**) Plots showing fold enrichment for association to CAD in a given -log_10_ P-value bin as a function of association with migraine.
(PDF)

S1 Table SNPs showing suggestive evidence (conjunctional FDR < 0.1) for shared association to migraine and coronary artery disease (CAD). SNP, Single nucleotide polymorphism; Chr, Chromosome; CAD, Coronary artery disease; FDR, False discovery rate. SE, standard error. na, SNP not available for analysis. *Positions refer to build NCBI36/hg18. †Conjunctional FDR < 0.01.
(DOC)

S2 Table Previously reported genome-wide significant associations in LD (*r*^*2*^ > 0.1) with the identified cross-phenotype loci. LD calculations are based on the European CEU population in the Phase 3 of the 1000 Genomes Project, as implemented in LDlink [30]. Reported associations are taken from the NHGRI GWAS catalog [31]. * PubMed ID for publication(s).
(DOC)
