# Supplementary figures and images for: Shared genetic risk between migraine and coronary artery disease: A genome-wide analysis of common variants
Source: PLoS One. 2017 Sep 28;12(9):e0185663. doi: 10.1371/journal.pone.0185663 (PMC5619824; doi:10.1371/journal.pone.0185663)

**a**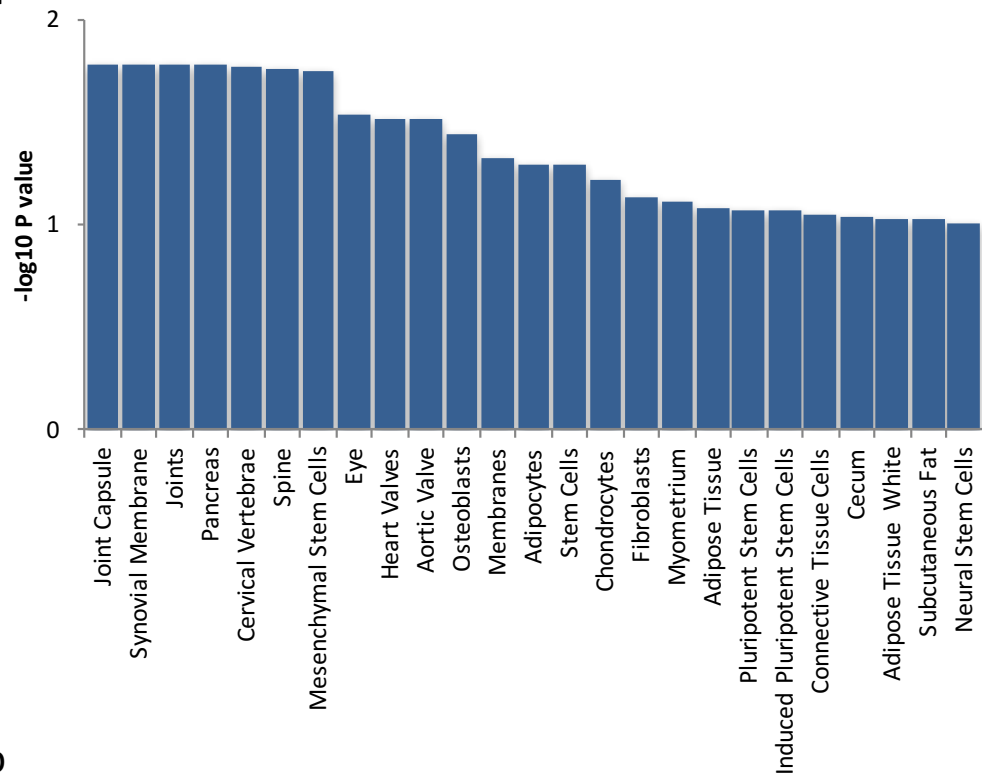**b**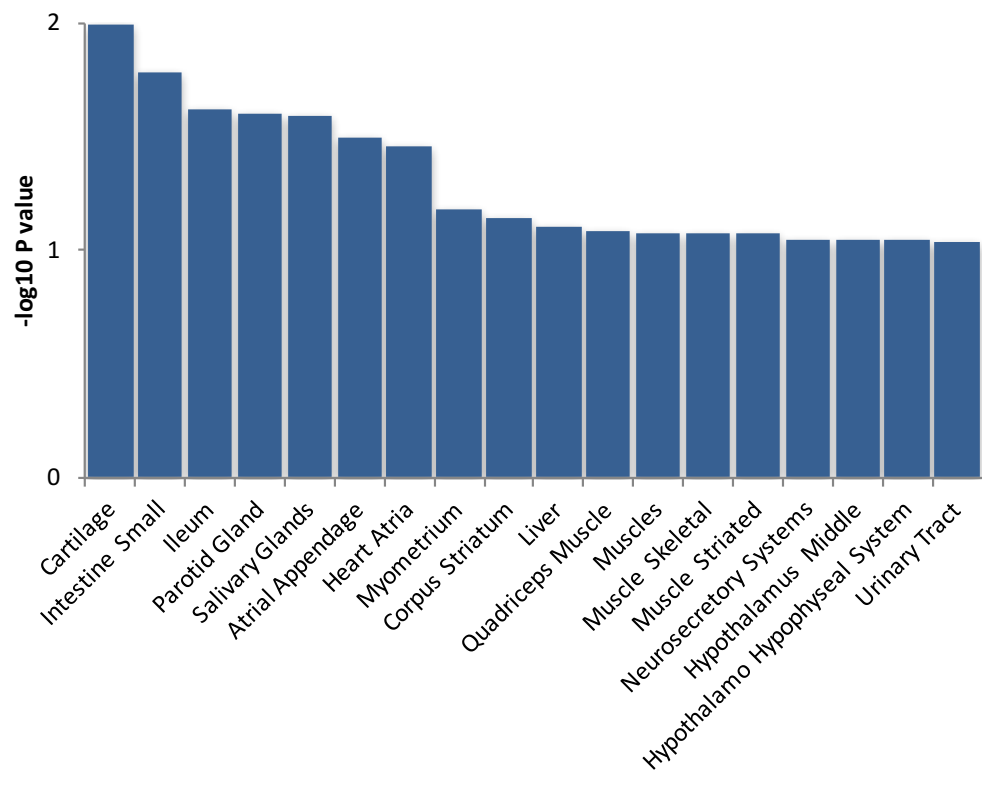

Supplement: S1 Fig — The analysis was based on expression data obtained from 37,427 human microarray samples for 209 tissue or cell types, as implemented in DEPICT [28]. Genes in loci with conjuctional FDR < 0.4 for cross-phenotype association between migraine and CAD were assessed for high expression in each of the annotation categories. The figures show the most enriched tissue types for the comparison of migraine against CARDIoGRAM (a) and for migraine against C4D (b). No tissue type was significantly enriched (false discovery rate <0.05) after controlling for multiple testing. (PDF) [file pone.0185663.s001.pdf]

**a**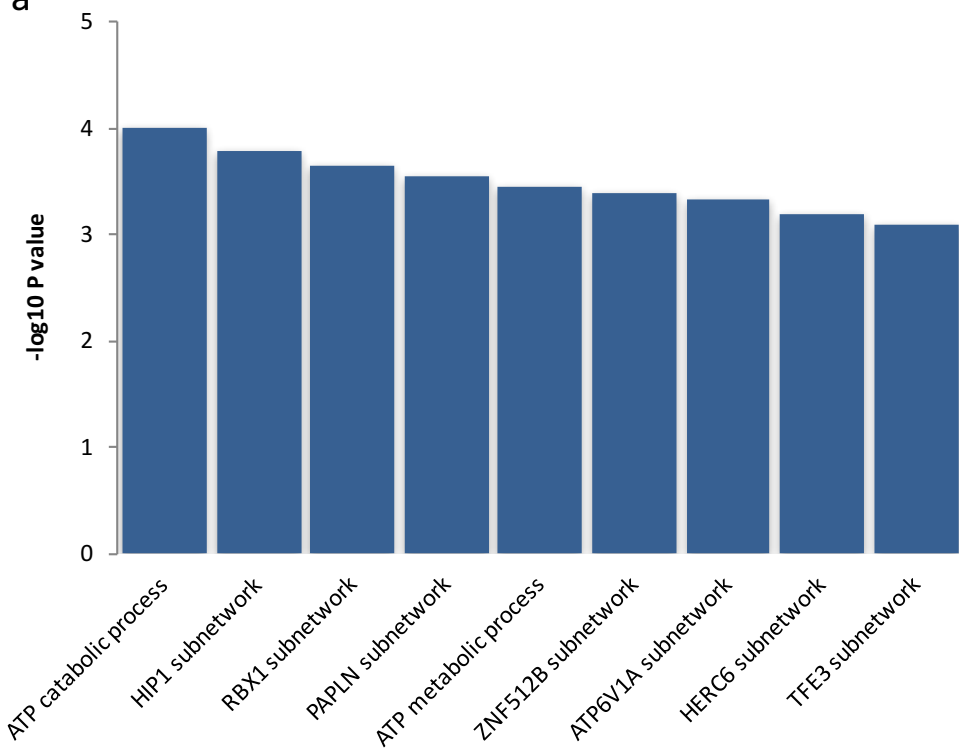**b**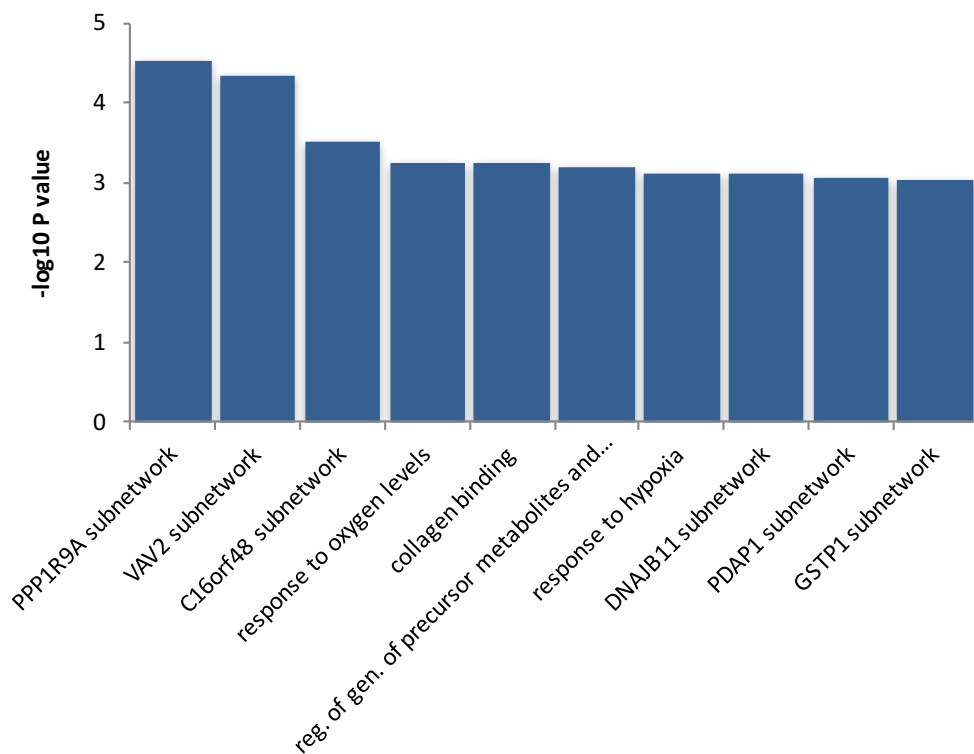

Supplement: S2 Fig — DEPICT reconstituted gene sets showing strongest evidence for enrichment for genes in loci with conjuctional FDR < 0.4 for cross-phenotype association between migraine against CARDIoGRAM (a) and for migraine against C4D (b). No reconstituted gene sets was significantly enriched (false discovery rate <0.05) after controlling for multiple testing. (PDF) [file pone.0185663.s002.pdf]

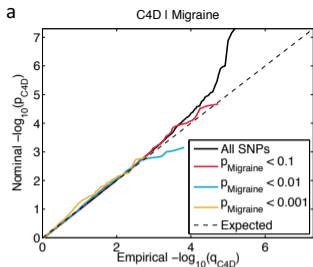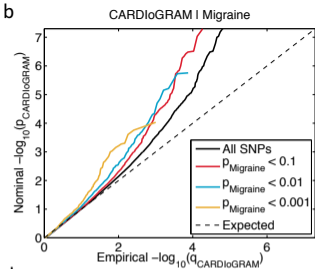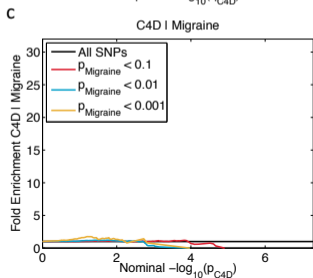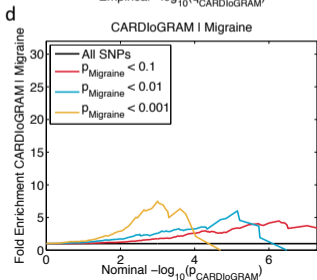

Supplement: S3 Fig — Genome-wide analysis after excluding all SNPs in PHACTR1 as well as any SNPs in linkage disequilibrium (r2 > 0.1) with these. (a-b) Conditional Q-Q plot of nominal versus empirical -log10 P-values (corrected for inflation) in CAD as a function of significance of association with migraine at the level of P ≤ 1, P < 0.1, P < 0.01 and P < 0.001. Dotted lines indicate the null-hypothesis. (c-d) Plots showing fold enrichment for association to CAD in a given -log10 P-value bin as a function of association with migraine. (PDF) [file pone.0185663.s003.pdf]
