# Supplementary material for: Shared genetic risk between migraine and coronary artery disease: A genome-wide analysis of common variants
Source: PLoS One. 2017 Sep 28;12(9):e0185663. doi: 10.1371/journal.pone.0185663 (PMC5619824; doi:10.1371/journal.pone.0185663)
Supplement: S1 Table — SNP, Single nucleotide polymorphism; Chr, Chromosome; CAD, Coronary artery disease; FDR, False discovery rate. SE, standard error. na, SNP not available for analysis. *Positions refer to build NCBI36/hg18. †Conjunctional FDR < 0.01. (DOC) [file pone.0185663.s004.doc]

| **Locus** | **SNP** | **Chr** | **Position*** | **Nearest Gene** | **Effect allele** | **Migraine beta (SE)** | **Migraine P-value** | **CAD**  **beta (SE)** | **CAD**  **P-value** | **Conjunctional FDR** |
| --- | --- | --- | --- | --- | --- | --- | --- | --- | --- | --- |
| Comparison Migraine and CAD: C4D | | | | |  |  |  |  |  |  |
| Locus 1 | rs9349379 | 6 | 13011943 | *PHACTR1* | A | 0.073 (0.014) | 6.44E-08 | -0.159 (0.017) | 6.50E-21 | 3.50E-05† |
| Locus 2 | rs733701 | 6 | 39279840 | *KCNK5* | T | 0.058 (0.014) | 2.24E-05 | 0.075 (0.019) | 5.86E-5 | 0.021† |
| Locus 3 | rs10786719 | 10 | 104627982 | *AS3MT* | A | na | na | na | na | na |
| Locus 4 | rs11065884 | 12 | 111818701 | *FAM109A* | A | na | na | na | na | na |
| Locus 5 | rs2161648 | 16 | 75314629 | *BCAR1* | G | na | na | na | na | na |
| Locus 6 | rs1889276 | 6 | 72330088 | *C6orf155* | C | 0.048 (0.012) | 4.60E-05 | 0.025 (0.017) | 0.15 | 0.80 |
| Comparison Migraine and CAD: CARDIoGRAM | | | | |  |  |  |  |  |  |
| Locus 1 | rs9349379 | 6 | 13011943 | *PHACTR1* | A | 0.083 (0.014) | 7.23E-09 | -0.093 (0.018) | 1.54E-7 | 3.90E-05† |
| Locus 2 | rs733701 | 6 | 39279840 | *KCNK5* | T | 0.057 (0.014) | 7.10E-05 | 0.057 (0.016) | 4.00E-04 | 0.065† |
| Locus 3 | rs10786719 | 10 | 104627982 | *AS3MT* | A | -0.048 (0.013) | 1.49E-04 | 0.052 (0.014) | 2.08E-4 | 0. 044† |
| Locus 4 | rs11065884 | 12 | 111818701 | *FAM109A* | A | 0.060 (0.015) | 8.40E-05 | -0.061 (0.017) | 3.30E-04 | 0.055† |
| Locus 5 | rs2161648 | 16 | 75314629 | *BCAR1* | G | 0.046 (0.013) | 3.10E-04 | -0.054 (0.014) | 1.30E-04 | 0.086† |
| Locus 6 | rs1889276 | 6 | 72330088 | *C6orf155* | C | 0.045 (0.012) | 3.00E-04 | 0.049 (0.014) | 5.40E-04 | 0.087† |
