# Supplementary material for: Shared genetic risk between migraine and coronary artery disease: A genome-wide analysis of common variants
Source: PLoS One. 2017 Sep 28;12(9):e0185663. doi: 10.1371/journal.pone.0185663 (PMC5619824; doi:10.1371/journal.pone.0185663)
Supplement: S2 Table — LD calculations are based on the European CEU population in the Phase 3 of the 1000 Genomes Project, as implemented in LDlink [30]. Reported associations are taken from the NHGRI GWAS catalog [31]. * PubMed ID for publication(s). (DOC) [file pone.0185663.s005.doc]

| **Locus** | **Index SNP (Gene)** | **Proxy SNP** | ***r^2^*** | **D’** | **Mapped Gene** | **Associated phenotype(s) (reported P-value)** | Reference(s)* |
| --- | --- | --- | --- | --- | --- | --- | --- |
| 1 | rs9349379 (PHACTR1) |  |  |  |  |  |  |
|  |  | rs9349379 | 1.0 | 1.0 | *PHACTR1* | Coronary heart disease (P=9.0E-26); Coronary artery calcification (P=4.0E-22); Migraine (P=3.0E-08); Migraine without aura (P=3.0E-10); Cervical artery dissection (P=1.0E-11); Myocardial infarction (P=9.0E-35); Coronary artery disease (P=2.0E-42) | 21378988, 21846871, 22144573, 22683712, 22745674, 22751097, 23793025, 25420145, 26343387 |
|  |  | rs9369640 | 0.372 | 1.0 | *PHACTR1* | Coronary artery disease (P= 3.0E-11) | 24262325 |
|  |  | rs4714955 | 0.349 | 1.0 | *PHACTR1* | Coronary artery disease or ischemic stroke, combined (P=4.0E-11) | 24262325 |
|  |  | rs12526453 | 0.323 | 0.963 | *PHACTR1* | Myocardial infarction (early onset) (P=1.0E-09); Coronary heart disease (P=1.0E-09) | 19198609, 21378990 |
| 2 | rs733701 (KCNK5) | rs10456100 | 0.868 | 0.971 | *KCNK5* | Migraine (6.9E-13) |  |
| 3 | rs10786719 (AS3MT) |  |  |  |  |  |  |
|  |  | rs7914558 | 0.979 | 1 | *CNNM2* | Schizophrenia (P=2.0E-08); Autism spectrum disorder, attention deficit-hyperactivity disorder, bipolar disorder, major depressive disorder, and schizophrenia, combined (P=2.0E-09) | 21926974, 23453885 |
|  |  | rs11191419 | 0.638 | 0.923 | *PTGES3P4 - C10orf32* | Schizophrenia (P=6.0E-19, P=3.0E-18) | 25056061, 26198764 |
|  |  | rs7085104 | 0.638 | 0.923 | *C10orf32-ASMT* | Schizophrenia (P=4.0E-13) | 23974872 |
|  |  | rs9527 | 0.46 | 0.934 | *C10orf32-ASMT* | Arsenic metabolism (P=3.0E-09) | 22383894 |
|  |  | rs12416687 | 0.46 | 0.934 | *C10orf32-ASMT* | Blood pressure (P=4.0E-09) | 24954895 |
|  |  | rs7894407 | 0.213 | 0.808 | *PDCD11* | White matter hyperintensity burden (P=3.0E-08) | 25663218 |
|  |  | rs55833108 | 0.205 | 1 | *CNNM2* | Schizophrenia (P=1.0E-08) | 26198764 |
|  |  | rs4919694 | 0.17 | 1 | *CNNM2* | Arsenic metabolism (P=3.0E-08) | 22383894 |
|  |  | rs11191454 | 0.104 | 1 | *C10orf32-ASMT* | Autism spectrum disorder, attention deficit-hyperactivity disorder, bipolar disorder, major depressive disorder, and schizophrenia, combined (P=1.0E-08) | 23453885 |
|  |  | rs12413409 | 0.104 | 1 | *CNNM2* | Intracranial aneurysm (P=1.0E-09); Coronary heart disease (P=1.0E-09); Coronary artery disease or large artery stroke, combined (P=2.0E-08) | 20364137, 21378990, 24262325 |
|  |  | rs11191548 | 0.104 | 1 | *CNNM2* | Systolic blood pressure (P=7.0E-24, P=7.0E-26, P=4.0E-17, P=3.0E-09); Blood pressure (P=8.0E-11); Diastolic blood pressure (P=7.0E-12) | 19430483, 21909110, 21909115, 21572416, 26390057 |
|  |  | rs11191560 | 0.104 | 1 | *NT5C2* | Body mass index (P=8.0E-09) | 25673413 |
|  |  | rs10883832 | 0.104 | 1 | *NT5C2* | Schizophrenia (P=2.0E-16) | 26198764 |
|  |  | rs11191580 | 0.104 | 1 | *NT5C2* | Schizophrenia (P=3.0E-08, P=2.0E-09); Autism spectrum disorder, attention deficit-hyperactivity disorder, bipolar disorder, major depressive disorder, and schizophrenia, combined (P=1.0E-08); Body mass index (P=4.0E-08) | 21926974, 22688191, 23453885, 24861553 |
|  |  | rs11191593 | 0.104 | 1 | *NT5C2* | Blood pressure (P=1.0E-15) | 21909110 |
